# Antitumor Efficacy of Liposome-Encapsulated NVP-BEZ235 Combined with Irreversible Electroporation for Head and Neck Cancer

**DOI:** 10.3390/molecules24193560

**Published:** 2019-10-01

**Authors:** Li Tian, Lucas Wang, Yang Qiao, Linfeng Lu, Patrick Lee, Ashley Chang, Saisree Ravi, Thomas A. Rogers, Marites P. Melancon

**Affiliations:** 1 Department of Interventional Radiology, Division of Diagnostic Imaging, The University of Texas MD Anderson Cancer Center, Houston, TX 77005, USA; Li.Tian.UU@gmail.com (L.T.); john.yang.qiao@gmail.com (Y.Q.); linfengl@bcm.edu (L.L.); 2 The University of Texas at Austin Dell Medical School, Austin, TX 78701, USA; lucaswang@utexas.edu; 3 College of Medicine, State University of New York Upstate Medical University, Syracuse, NY 13421, USA; PATRICK.LEE829@gmail.com; 4 McGovern Medical School, The University of Texas Health Science Center at Houston, Houston, TX 77030, USA; Ashley.Chang@uth.tmc.edu; 5 Department of BioSciences, Rice University, Houston, TX 77251, USA; sr56@rice.edu; 6 Department of Chemistry, Mississippi State University, Starkville, MS 39762, USA; Talexrogers@yahoo.com; 7 Graduate School of Biomedical Sciences, The University of Texas Health Science Center at Houston, Houston, TX 77030, USA

**Keywords:** nanoparticles, liposomes, irreversible electroporation, combination therapy, cancer, head and neck cancer, drug delivery, NVP-BEZ235

## Abstract

Irreversible electroporation (IRE) kills tumor cells by the delivery of short pulses of strong electric fields. However, the field strength decreases with distance from the treatment center. When IRE cannot eradicate the entire tumor mass, the surviving tumor cells can regrow. NVP-BEZ235 is a dual PI3K/mTOR inhibitor that has been administered orally in clinical trials. However, its hydrophobicity and poor water solubility make NVP-BEZ235 difficult to deliver to target areas. To improve its pharmacokinetics and therapeutic efficacy, we have encapsulated NVP-BEZ235 in a liposome (termed as L-BEZ). Our current study focuses on the long-term antitumor efficacy of IRE and intratumoral injection of L-BEZ in HN5 head and neck cancer xenografts in nude mice. We compared in vitro efficacy, as well as the effect on tumor size and growth rate in vivo, between IRE alone, IRE + oral BEZ, and IRE + L-BEZ over the course of two months. All animals in the control group were sacrificed by day 36, due to excess tumor burden. Tumors treated with IRE alone grew faster and larger than those in the control group. IRE + oral BEZ suppressed tumor growth, but the growth rate increased to that of the controls toward the end of 21 days. Only IRE + L-BEZ eradicated the tumor masses, with no palpable or extractable tumor mass observed after two months. The combination of IRE and L-BEZ could effectively eradicate tumors and prevent recurrence.

## 1. Introduction

In 2019, an estimated 65,410 people will develop head and neck cancer and 14,620 will die from it in the United States [1]. Local or regional recurrence of head and neck cancer as high as 50% has been observed [2]. Squamous cells are the most common head and neck cancer cell type. The principal treatment of locoregionally advanced squamous cell head and neck carcinoma is gradually changing from surgery to other treatment modalities [3]. Combined modality approaches (surgery, radiotherapy (RT), and chemotherapy) are generally required to optimize the chances for long-term disease control. These combined modality approaches include primary surgery followed by either postoperative RT or concurrent chemoradiotherapy, as well as induction chemotherapy (the addition of chemotherapy prior to surgery and RT), concurrent chemoradiotherapy without surgery, and sequential therapy (induction chemotherapy followed by concurrent chemoradiotherapy) without surgery [4]. However, the toxicity associated with treatment for head and neck cancer (whether surgery, RT, or chemotherapy) is substantial, and every effort should be made to minimize side effects and treat these complications. Thus, new treatment modalities are emerging to minimize toxic injuries to the surrounding tissues. One such modality is irreversible electroporation (IRE), because of its advantages in treating cutaneous and subcutaneous tumors while preserving extracellular matrix, blood flow, and nerves [5]. 

IRE is an emerging treatment modality that delivers short pulses of strong electric fields to create membrane defects and kill tumors [6,7,8]. However, the electric field in the treatment area is strongest at the treatment center, nearest the electrodes, and weakens as the distance from the treatment center increases [9,10]. The margins of the tumor receive the weakest electric field. When the electric field is weaker than 1000 V/cm, the procedure is considered reversible electroporation (RE) instead of IRE, as the cell membrane reseals after the electroporation, and the cells survive. A tumor may receive IRE to the treatment center, but RE at the margins. Furthermore, our previous results in Hep3B hepatocellular carcinoma cells suggest that RE leads to greater viability of cancer cells in vitro and to higher proliferation, as shown in the increased Ki67 staining of the tumor margin in vivo [11]. Clinical results also suggest that more than 10% of local tumor recurrences occur at the ablated site for patients treated with IRE [12]. However, this weakened electric field, or RE, also presents an opportunity to co-deliver therapeutic agents, such as nanoparticles.

Nanoparticles are a versatile tool in cancer treatment [13]: they can be designed from various materials and into various structures and shapes, and they can carry various therapeutic agents and targeting moieties. Liposomes were selected for our study, not only because they can carry hydrophobic drugs in their lipid layers, but also because of their structural resemblance to the cell membrane—since electroporation can disrupt the cell membrane, it should also be able to disrupt the liposomal membrane [14]. Our previous results have shown that co-delivery of a single dose of liposomal NVP-BEZ235 (L-BEZ) with IRE can decrease the proliferation of IRE-treated tumor masses [13]. However, one dose of co-delivery of L-BEZ was not able to eradicate the tumors. Therefore, an aim of our current research is the optimization of this combination treatment mechanism. 

PI3K inhibitors can enhance the radiosensitivity of squamous cell head and neck carcinoma [15,16]. The dual PI3K/mTOR inhibitor NVP-BEZ235 (BEZ) [17] has been shown to enhance the radiosensitivity of SQ20B laryngeal and FaDu hypopharyngeal cancer cells, overexpressing epidermal growth factor receptor (EGFR) [18]. However, BEZ is highly hydrophobic and poorly water soluble, which makes it difficult to deliver into the target areas. In both preclinical studies [19] and clinical trials (NCT00620594 and NCT01856101), BEZ was given orally at a high dose over a long period: 21 days in the preclinical studies and daily for 28 days per cycle in the clinical trials. However, the preclinical data showed a low plasma concentration of BEZ, which suggests a very low bioavailability. To circumvent this low bioavailability, BEZ was encapsulated into a liposomal formulation for direct intratumoral injection. This intratumoral injection was compared with the oral BEZ administration.

In our current study, we tested the long-term efficacy of combined IRE and L-BEZ in HN5 head and neck cancer xenografts in mice. We compared the effects of IRE alone, combined IRE and oral BEZ, and combined IRE and L-BEZ in vitro and in vivo over the course of two months. Our results show that there is complete tumor regression with IRE + L-BEZ compared to the other groups tested, showing the efficacy of this treatment combination for head and neck cancer.

## 2. Results

### 2.1. Synthesis and Characterization of Liposomal NVP-BEZ235

Table 1 shows the liposome formulation, fabrication method, BEZ concentration in the resulting liposomes, and the loading efficiency. Z-average particle size was used, and reported in Table 1 as mean ± SD. The particle size was measured from each liposome preparation. Extrusion of liposomes through a 100 nm membrane was used to produce particle sizes smaller than 100 nm with a polydispersity index below 0.2. Extrusion also yielded lower loading efficiency, resulting in low BEZ encapsulation concentration compared with that of liposomes that were not extruded. Liposomes obtained without extrusion had a larger particle size, a higher BEZ encapsulation efficiency, and a higher BEZ concentration. Unextruded liposomes were used in the in vitro cytotoxicity study and the in vivo study, owing to their high drug loading. 

### 2.2. Effect of Electroporation Field Strength on Liposomal NVP-BEZ235 Stability

**Figure 1:**
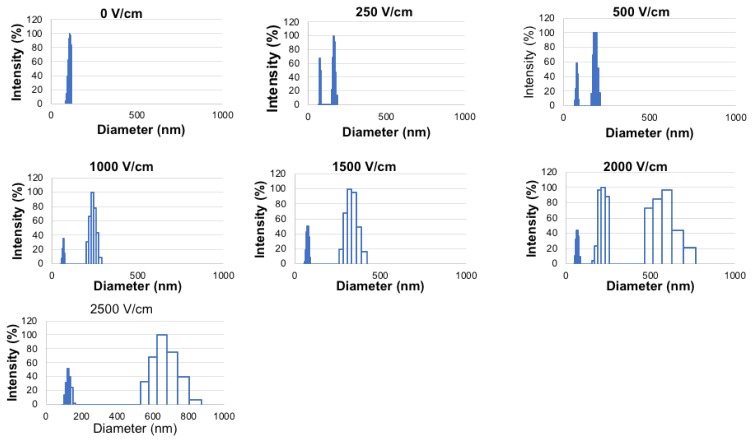
Liposomes were disrupted by electroporation as low as 250 V/cm.

The effect of electroporation field strength on L-BEZ was examined. Extruded L-BEZs were used for visualization purposes, because the unextruded L-BEZs had a large initial particle size, disguising the sizes of disrupted liposomes. The extruded L-BEZs showed a small size and a narrow particle size distribution before electroporation. When the electroporation field strength was as low as 250 V/cm, L-BEZ started to exhibit two particle size distributions: one distribution was larger than the original particle size, and one was smaller, which indicated the destruction of L-BEZ at 250 V/cm. At 2000 V/cm, more than two particle size distributions were observed. The higher the field strength was, the higher the polydispersity index, as is shown from the wider particle size distribution; this suggests that the electroporated L-BEZ had a wider particle size distribution. The sizes of the largest particles also generally increased with increasing field strength. These results suggest that electroporation disrupts the liposomes over a wide range of field strengths (Figure 1). 

### 2.3. Liposomal NVP-BEZ235 Cytotoxicity against HN5 Cells

**Figure 2:**
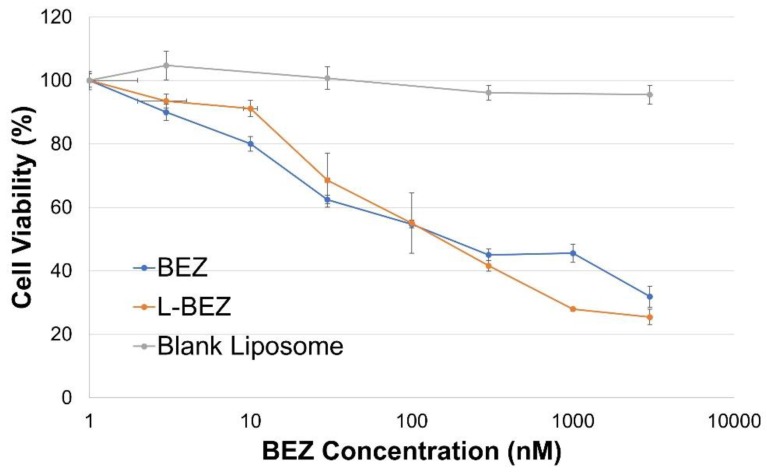
Cytotoxicity of L-BEZ against HN5 head and neck cancer cells after 72 h of incubation. L-BEZ and BEZ alone had similar IC50 values.

The cytotoxicity of L-BEZ against HN5 cells was compared with that of BEZ alone, as well as that of blank liposomes. HN5 cell viability decreased with increasing BEZ concentration for both BEZ alone and L-BEZ (Figure 2). Liposome encapsulation of BEZ did not affect its cytotoxicity against the HN5 cell line; L-BEZ and BEZ alone had similar IC50 values of 175.2 nM and 197.2 nM, respectively. Blank liposomes had little cytotoxicity. 

### 2.4. Effect of Electroporation at Different Field Strengths on HN5 Cells

**Figure 3:**
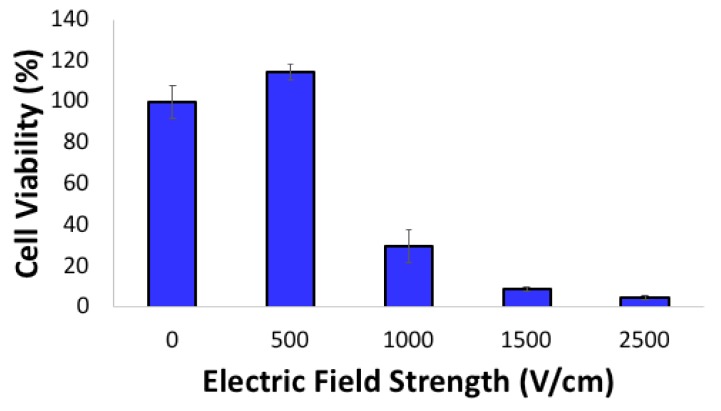
Cytotoxicity of electroporation at different field strengths against HN5 cells 72 h after treatment. Irreversible electroporation (IRE) (≥1000 V/cm) decreased cell viability, while reversible electroporation (RE) (<1000 V/cm) increased cell viability.

The effect of electroporation on HN5 cells was assessed (Figure 3). Increasing the electroporation field strength decreased the cell viability of HN5: viability decreased to 30% at 1000 V/cm and to 4.5% at 2500 V/cm (Figure 3). However, 72 h after electroporation at 500 V/cm, the viability of HN5 cells increased to 114%.

### 2.5. Effect of Reversible Electroporation, NVP-BEZ235, Liposomal NVP-BEZ235, and Their Combinations on HN5 Cells

**Figure 4:**
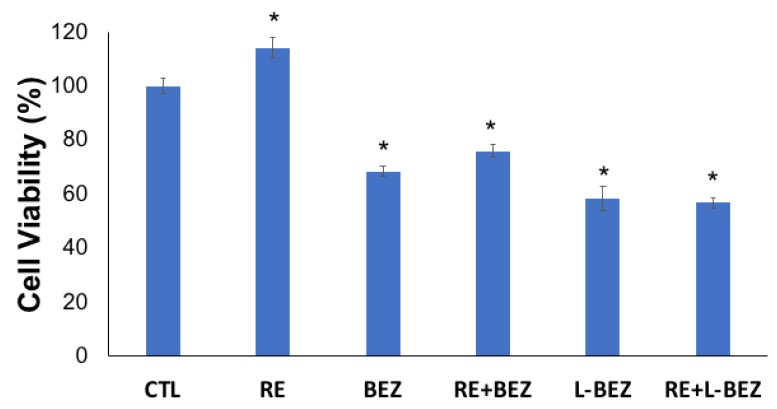
Effect of RE alone or in combination with NVP-BEZ235 (BEZ) or L-BEZ on HN5 cells. RE increased cell viability (114.3 ± 3.9), but its combination with either BEZ (76.1 ± 2.4) or L-BEZ (56.8 ± 2.1) significantly decreased cell viability, as did BEZ alone (68.8 ± 2.0) or L-BEZ alone (58.5 ± 4.4) when compared with the control, using one-way analysis of variance followed by the Holm–Sidak method (* *p* < 0.05). CTL: control.

The cytotoxic effect of BEZ with and without liposomes, as well as in combination with low-field-strength electroporation, was assessed. A low field strength of 500 V/cm was chosen because this power had shown no cytotoxicity (Figure 3), so any decrease in cell viability would be due primarily to the drug or its carrier. A BEZ concentration of 30 nM, lower than the IC50 values, was chosen. Although RE increased cell viability 72 h after treatment, the combination of RE with either L-BEZ or BEZ alone significantly decreased cell viability (Figure 4). The cell viability levels after RE, BEZ, RE + BEZ, L-BEZ, and RE + L-BEZ groups were 114.3 ± 3.9%, 68.5 ± 2.0%, 76.1 ± 2.4%, 58.5 ± 4.4%, and 56.8 ± 2.1%, respectively. The cell viability of the RE group was significantly higher than that of all the other treatment groups (*p* < 0.001; one-way analysis of variance). The BEZ and RE + BEZ groups had similar levels of cell viability, as did the L-BEZ and RE + L-BEZ groups. These results suggest that the combination of RE and the chemotherapy agent behaves similarly to monotherapy in vitro 72 h after treatment. Statistics was conducted by one-way ANOVA, followed by the Holm–Sidak method. The *p* values are all <0.001 for RE vs. RE + L-BEZ, RE vs. L-BEZ, RE vs. BEZ, RE vs. RE + BEZ, RE + BEZ vs. RE + L-BEZ, RE + BEZ vs. L-BEZ, BEZ vs. RE + L-BEZ, and BEZ vs. L-BEZ. The *p* value of RE + BEZ vs. BEZ was 0.002. The *p* value of L-BEZ vs. RE + L-BEZ was 0.386. 

### 2.6. In Vivo Efficacy of Irreversible Electroporation Plus Liposomal NVP-BEZ235

**Figure 5:**
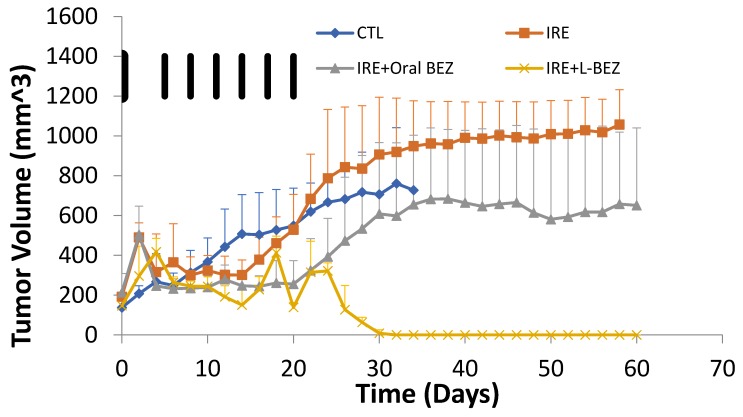
Tumor growth over the course of 60 days (*n* = 4) in nude mice bearing HN5 xenografts. The black bars represent the days when L-BEZ was injected. IRE suppressed tumor growth initially, but the growth eventually exceeded that of the control (CTL) group. IRE + oral BEZ suppressed tumor growth longer than IRE alone, but treated tumors eventually recurred in both groups. IRE + L-BEZ effectively suppressed tumor growth and eventually eradicated the tumors; no palpable tumor was observed in the treated animals after 30 days.

The efficacy of IRE + L-BEZ was evaluated in nude mice bearing HN5 xenografts (Figure 5). The animals in the control group were euthanized earlier than the treated animals, because of excessive tumor burden. The average tumor size in the IRE group remained stable for about two weeks, and then increased drastically and was bigger than that in the control group at the end of one month. The tumor growth of the IRE + oral BEZ group was suppressed for about three weeks, which was also the duration of oral BEZ administration; then, the tumors grew rapidly. The tumors in the IRE + L-BEZ group disappeared after the first month of treatment. The fluctuations in tumor size within 21 days in the IRE + L-BEZ group were likely due to the volume introduced by intratumoral injections, not tumor growth. No palpable tumor was found after 30 days of treatment in the IRE + L-BEZ group. All animals in the control group were sacrificed by Day 36, due to excess tumor burden. 

**Figure 6:**
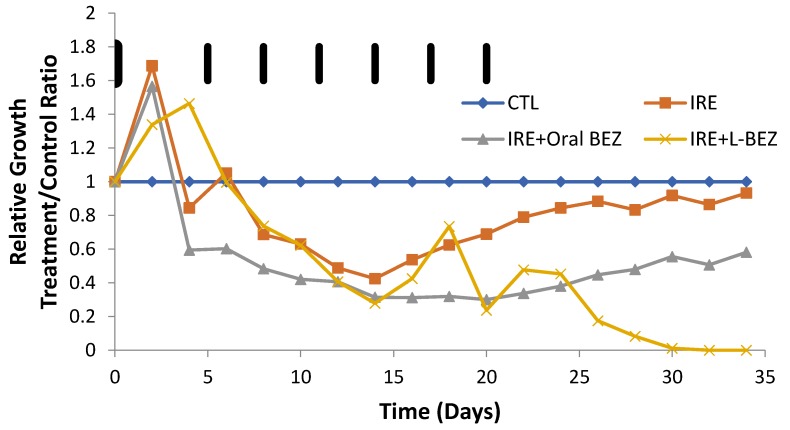
Relative tumor growth curve as the ratio of mean tumor size between treatment and control (CTL) groups (*n* = 4). The black bars represent the days when L-BEZ was injected. IRE decreased relative tumor growth for two weeks, after which the tumor growth exceeded that of the control group. At the end of one month, the tumor growth rate of the IRE and control groups were similar. The combination of IRE with oral BEZ (daily for 21 days) suppressed tumor growth longer. However, toward the end of 21 days, the tumor growth rate was similar to that of the CTL group, suggesting that the effect of oral BEZ was saturated. As soon as oral BEZ was stopped, the tumor growth rate exceeded that of the control group. A combination with L-BEZ effectively eradicated the treated tumors, which continued to shrink even after the last dose of L-BEZ. No palpable tumor was observed by the end of one month. The fluctuation in relative tumor growth in the IRE + L-BEZ group was caused by the volume introduced by the intratumoral injection of L-BEZ.

Another way to look at tumor volume over time is to plot the relative growth in each group over time. Relative growth is the ratio of the mean tumor size in the treatment group divided by that in the control group, or the T/C ratio (Figure 6). The control group size was divided by itself (control/control), so its relative growth value remained at 1 with a slope of 0. The slopes for the three treatment groups show the tumor growth rates in relation to that of the control group (Figure 5). Initially, the treated tumors grew faster than those of the controls before day 5 and started to decrease around day 6, when BEZ treatment started. On day 14, the relative growth of the tumors in the IRE group started to increase, and at day 32, the tumor growth rate in the IRE group was almost the same as that in the control group. The IRE + oral BEZ group also showed a decrease in relative tumor growth rate before day 14, followed by an increase in relative growth. The combination with oral BEZ (daily for 21 days) suppressed tumor re-growth longer compared with IRE alone. However, as soon as oral BEZ was stopped at 21 days, the relative tumor growth rate exceeded that of the control group. Finally, the combination of IRE with L-BEZ completely and effectively eradicated the treated tumors, which continued to shrink even after the last dose of L-BEZ. No palpable tumor was observed in this group by the end of one month. The fluctuation in the T/C ratio in the IRE + L-BEZ group was likely caused by the volume introduced by the intratumoral injection of L-BEZ. 

**Figure 7:**
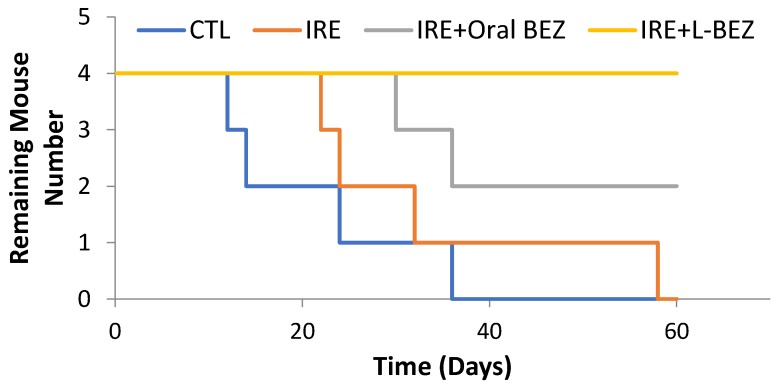
Survival curves over the course of 60 days (*n* = 4). All animals in the control (CTL) group were euthanized by day 36. Only one animal in the IRE group survived until day 58. Half the animals in the IRE + oral BEZ group survived through day 60, but both had tumors. All animals in the IRE + L-BEZ group survived tumor-free through day 60.

**Figure 8:**
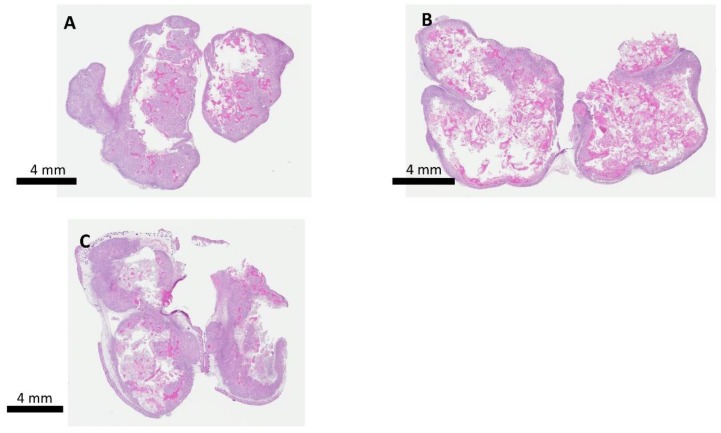
Hematoxylin and eosin staining of tumor masses at the end of two months. (**A**) CTL, (**B**) IRE, and (**C**) IRE + oral BEZ groups. Tumors extracted showed massive necrosis at the center of the tumors. There was no extractable tumor in the IRE + L-BEZ group.

Figure 7 shows the survival curves of all the study groups. All animals in the control group were euthanized by day 36, owing to the tumor burden exceeding the allowable limit for mice. Three out of four animals in IRE group were euthanized by day 32, and one survived until Day 58. Half the animals in the IRE + oral BEZ group survived until day 60, but both continued to have very large tumors. All animals in the IRE + L-BEZ group survived tumor-free through Day 60. Hematoxylin and eosin staining of the tumors in the control, IRE, and IRE + oral BEZ groups at the end of two months showed necrosis at the center of the tumors, due to prolonged tumor growth (Figure 8). No extractable tumor mass was found in the IRE + L-BEZ group upon euthanasia.

## 3. Discussion

IRE provides a safer, faster, and more effective means to treat tumors compared to other ablative therapies. Studies have shown that IRE was able ablate tissues close to the blood vessels without harming them [20]. IRE also has a unique selectivity—for example, Onik et al. were able to destroy cellular components of prostate tissue while preserving the collagen tissue intact underneath [21]. Thus, IRE could be beneficial for tumors, such as head and neck cancers, that are located in close proximity to critical structures, including nerves and blood vessels. However, it has been shown in various studies that incomplete IRE could enhance the growth of tumors, and thus combining it with a chemotherapeutic drug could lead to the complete eradication of the disease [8,22,23]. DPPC (1,2-dipalmitoyl-sn-glycero-3-phosphocholine) and DSPE-PEG (1,2-distearoyl-sn-glycero-3-phosphoethanolamine-*N*-[amino(polyethylene glycol)-2000] (ammonium salt)) were used in the liposome formulation because they are among the most studied lipids, as well as well-established liposome components [14,24,25,26]. Our previous study also used DPPC and DSPE-PEG liposomes encapsulating BEZ. Their combination with IRE showed antiproliferation efficacy on Hep3B xenografts after a single treatment [11].

In our current study, we tested the long-term efficacy of combined IRE and L-BEZ in HN5 head and neck cancer xenografts in mice. We compared the effects of IRE alone, combined IRE and oral BEZ, and combined IRE and L-BEZ in vitro and in vivo over the course of two months. We found that the tumors treated with monotherapy IRE grew faster and larger than did those in the control group. IRE + oral BEZ suppressed tumor growth, but the growth rate increased to that of the controls toward the end of 21 days. Only IRE + L-BEZ eradicated the tumor masses, with no palpable or extractable tumor mass observed after 2 months.

The formation of an aggregate structure has previously been observed when the PEG-phospholipid ratio exceeded 10 mole percent. When the polyethylene glycol (PEG)/phospholipid ratio exceeded 15 mole percent, mixed micelles, not liposomes, were formed [27]. Similarly, we found that then DSPE-PEG reached 15 mole percent, the extrusion pressure was extremely high; thus, we kept the DSPE-PEG ratio at 5 mole percent. Extrusion yielded smaller particles with narrower size distributions, but also decreased the BEZ loading efficiency to a concentration that was too low to be administered to the animals. Therefore, L-BEZ obtained without extrusion was used in in vitro and in vivo experiments.

Our examination of the effect of electroporation on L-BEZ stability indicates that electroporation can destroy L-BEZs at a field strength as low as 250 V/cm. L-BEZ destabilization also occurred over a wide range of electroporation field strengths. The cytotoxicity results also suggested that encapsulation of BEZ into the liposomes did not affect its ability to destabilize HN5 cells in vitro.

Our previous in vitro results [11] for electroporation of Hep3B cells suggests that electroporation in the RE range leads to higher cell viability than does IRE. The same results were observed on HN5 cells (Figure 3). However, when we electroporated a non-cancerous, immortalized, human vascular endothelial cell line RF24 (Figure S1), no increased cell proliferation was observed. Altogether, these observations suggest that cancer cells that have undergone RE instead of IRE pose a risk of cancer recurrence. In our in vitro experiments, the combination of RE and the chemotherapeutic agent behaved like the chemotherapeutic drug alone at 72 h after treatment. On the other hand, in the in vivo experiments, conducted over a much longer time, the outcome in the group treated with IRE + L-BEZ suggested complete eradication and no recurrence. These results suggest that the combination of IRE with a proper anti-cancer drug formulation can decrease the risk of cancer recurrence.

Our in vivo results (Figure 4 and Figure 5) show that when tumor cells survive IRE, the tumor recurs, but the combination of IRE with an anti-cancer agent can provide a more complete therapeutic outcome. In the IRE group, after suppression of tumor growth for two weeks, a clear turning point was observed on day 14 (Figure 5), after which the tumors grew faster than those in the control group. Tumor growth was suppressed longer by the combination of IRE + oral BEZ, for three weeks. However, during the third week, despite smaller tumors than in the control group, the tumor growth rate in the IRE + oral BEZ group became similar to that in the control group. This finding suggests that at the end of one oral BEZ cycle, although the patient is still taking BEZ orally, oral BEZ may no longer inhibit tumor growth. This possible limit to the efficacy of oral BEZ could be one reason why patients have needed multiple oral BEZ cycles in clinical trials (NCT00620594 and NCT01856101), which may lead to adverse effects and drug resistance. In contrast, both the tumor size and growth rate in the IRE + L-BEZ group exhibited continual decreases. The initial tumor size increase in all IRE-treated groups within three days of the start of treatment was likely due to local swelling caused by IRE treatment, not real tumor growth. The 100% survival rate over the course of two months further supports the efficacy of IRE + L-BEZ. The IRE + L-BEZ group showed superior outcomes, even though the total administered amount of BEZ in the IRE + L-BEZ group was only 6.6% of that in the IRE + oral BEZ group. 

## 4. Materials and Methods

### 4.1. Chemicals

DPPC (1,2-dipalmitoyl-sn-glycero-3-phosphocholine) and DSPE-PEG (1,2-distearoyl-sn-glycero-3-phosphoethanolamine-*N*-[amino(polyethylene glycol)-2000] (ammonium salt)) were purchased from Avanti Polar Lipids (Alabaster, AL, United States) and used without further purification. BEZ was purchased from LC Laboratories (Woburn, MA, United States). All other chemicals were purchased from Sigma-Aldrich (St. Louis, MO, United States) and used without further purification. 

### 4.2. Liposome Preparation

Liposomes were prepared using the hydration–sonication method with an optional extrusion step [24]. Briefly, DPPC, DSPE-PEG, and BEZ were dissolved in a 4:1 mixture of chloroform to methanol (*v/v*), and vacuum-dried on a rotary evaporator at 49 °C to form a thin film. The thin film was then hydrated in 4-(2-hydroxyethyl)-1-piperazineethanesulfonic acid-buffered saline (HBS) solution. To obtain the liposome solution, 20 rounds of hydration–sonication cycles at 60 °C were performed. Free BEZ was removed by passing the liposome solution through a Sephadex G-25 column (GE Healthcare, Chicago, IL, United States), to obtain the final BEZ liposome solution (L-BEZ). Blank liposomes were prepared similarly, but without BEZ. Particle size was monitored by measuring dynamic light scattering at a scatter angle of 90°, using a Brookhaven Instruments particle-size analyzer (Holtsville, NY, United States). Z-average particle size was reported. All liposome solutions were stored at 4 °C and used within one week of production. 

### 4.3. NVP-BEZ235 Quantification

The amount of BEZ in L-BEZ was quantified on a CLARIOstar plate reader (BMG Labtech, Ortenberg, Germany), with the excitation wavelength at 325 nm and emission wavelength at 425 nm. BEZ was released from L-BEZ by adding two parts dimethyl sulfoxide (DMSO) to one part L-BEZ by volume. The mixture was mixed on a vortex and cooled to room temperature. The blank liposome was processed the same way, and used as a blank control. BEZ standards were prepared by dissolving BEZ in DMSO:HBS (2:1, *v/v*) at various concentrations. 

### 4.4. Cell Culture and Liposomal NVP-BEZ235 Cytotoxicity Assays

The head and neck squamous cell carcinoma cell line HN5, a gift from Dr. Jeffrey Myers’s laboratory, were cultivated in Dulbecco modified Eagle medium, supplemented with 10% fetal bovine serum (F0926, Sigma-Aldrich) and 1% penicillin–streptomycin (30-002-CI, Corning, Corning, NY, United States). Cell cultures were maintained at 37 °C and 5% CO_2_. For cytotoxicity studies, the cells were plated in 96-well plates. Twenty-four hours after the cells were plated, BEZ and L-BEZ were diluted in Roswell Park Memorial Institute medium, to various final concentrations, and added to the cells. After another 72 h of incubation, cytotoxicity was examined using a 3-(4,5-dimethylthiazolyl-2)-2,5-diphenyltetrazolium bromide (MTT) assay (M6494, Thermo Fisher Scientific, Waltham, MA, United States). Corresponding blank liposomes were also tested. The cell viability curve was plotted in Excel (Microsoft, Redmond, WA, United States), and the half-maximal inhibitory concentration (IC50) values were read from the curve.

### 4.5. In Vitro Electroporation and Cell Viability Assays

In vitro electroporation was carried out using a BTX ECM 830 electroporation system (Harvard Apparatus, Holliston, MA, United States). HN5 cells were subjected to electroporation at field strengths ranging from 0 to 2500 V/cm. The electroporated cells were then plated in 96-well plates. An MTT assay was conducted 72 h after electroporation to assess cell viability. 

For combination therapy with BEZ or L-BEZ, IRE was performed 90 s after the addition of BEZ or L-BEZ. Then an MTT assay was carried out 72 h later. 

### 4.6. Treatment of Mouse Xenograft Tumors with Irreversible Electroporation and NVP-BEZ235

We determined the acute effects of treatment with IRE in combination with L-BEZ or oral BEZ, using mice bearing HN5 xenografts. Female nude mice (nu/nu, 4 to 5 weeks old) were purchased from the Experimental Radiation Oncology Breeding Core at MD Anderson Cancer Center (Houston, TX, United States). The protocol was approved by the Institutional Animal Care and Use Committee (protocol number 00001234-RN01). Three million HN5 cells in phosphate-buffered saline:Matrigel (1:1; Corning) were inoculated on the right hind leg of each mouse. Treatment was started when the tumor size reached 8 mm. The mice were randomized into four treatment groups, with four mice per group: control (no treatment), IRE alone, IRE + oral BEZ, and IRE + L-BEZ. The treatment cycle was 21 days (from Day 0 to Day 20). Mice in the IRE group received a single dose of IRE at 2500 V/cm, with 99 pulses at 1-s intervals. The L-BEZ group received a single intratumoral injection of 100 μL of L-BEZ (equivalent to 0.1 mg of BEZ). The IRE + oral BEZ group received oral BEZ at 50 mg/kg of the body weight at Day 0. One hour after the oral BEZ administration, IRE was applied. Then, the animals were given oral gavage of BEZ daily until Day 20. The IRE + L-BEZ group was intratumorally injected with the equivalent of 0.1 mg of BEZ. Ninety seconds later, the animals received a single dose of IRE. On Day 5, the animals were intratumorally injected with 0.1 mg of BEZ, followed by one dose of intratumoral injection of 0.2 mg of BEZ on Days 8, 11, 14, 17, and 20. A total of seven injections were given. The animals were anesthetized with isoflurane for the IRE treatment and returned to their cages when they had fully recovered from the anesthesia and the IRE treatment. Tumor size and body weight were monitored every other day for two months. At the end of the two months, all animals in the four groups were killed via a CO_2_ chamber followed by cervical dislocation, and tumors were extracted. Hematoxylin and eosin staining was performed by the Department of Veterinary Medicine and Surgery at MD Anderson. The stained slides were scanned in Aperio eSlide Manager (Leica Biosystems, Wetzlar, Germany), and the percentage of necrotic cells was quantified using Aperio ImageScope software (Leica Biosystems). 

### 4.7. Statistical Analysis

One-way analysis of variance, followed by the Holm–Sidak method, was used to compare results between groups at the α = 0.05 level. SigmaPlot software (Systat Software, San Jose, CA, United States) and Excel (Microsoft) were used for statistical analysis. 

## 5. Conclusions

Cancer recurs when IRE does not eradicate all the tumor cells, and the growth speed of these recurring tumors exceeds that of untreated tumors, both in vitro and in vivo. Combination with a chemotherapeutic agent can enhance the antitumor efficacy of IRE. The effect of oral BEZ in combination with IRE reached a point of saturation after the equivalent of one oral BEZ cycle. This saturation could explain why oral BEZ was given to the animal for 21 days per cycle. The combination of chemotherapy with L-BEZ, however, effectively eradicated tumors and led to tumor-free survival after 60 days in all animals given this treatment combination. The combination of IRE and L-BEZ could effectively eradicate tumors and prevent tumor recurrence. Further preclinical and clinical studies of the combination of IRE and drug-loaded nanoparticles could be beneficial to cancer patients.

## Figures and Tables

**Table 1:** Liposome formulation, resulting liposomal NVP-BEZ235 (L-BEZ) concentration, and loading efficiency.

Lipid Composition (DPPC:DSPE-PEG Molar Ratio)	Total Lipid Weight (mg)	Added BEZ (mg)	Extrusion	Particle Size (nm)	Polydispersity	L-BEZ Concentration (mg/mL)	Loading Efficiency (%)
95:5	40	0.4	Y	71.6 ± 0.8	0.18	0.017	11%
90:10	40	0.4	Y	82.3 ± 0.8	0.18	0.017	11%
85:15	40	0.4	Y	90.5 ± 1.2	0.19	0.017	11%
95:5	80	0.8	Y	84.3 ± 1.8	0.17	0.029	9%
95:5	80	4	N	276.1 ± 40.2	0.28	1.16	73%
95:5	80	8	N	411.4 ± 75.4	0.36	2.42	75%

## Supplementary Materials

The Supplementary Materials are available online.
