# Supplementary material for: Antitumor Efficacy of Liposome-Encapsulated NVP-BEZ235 Combined with Irreversible Electroporation for Head and Neck Cancer
Source: Molecules. 2019 Oct 1;24(19):3560. doi: 10.3390/molecules24193560 (PMC6804038; doi:10.3390/molecules24193560)
Supplement: Supplementary file 1 [file molecules-24-03560-s001.pdf]

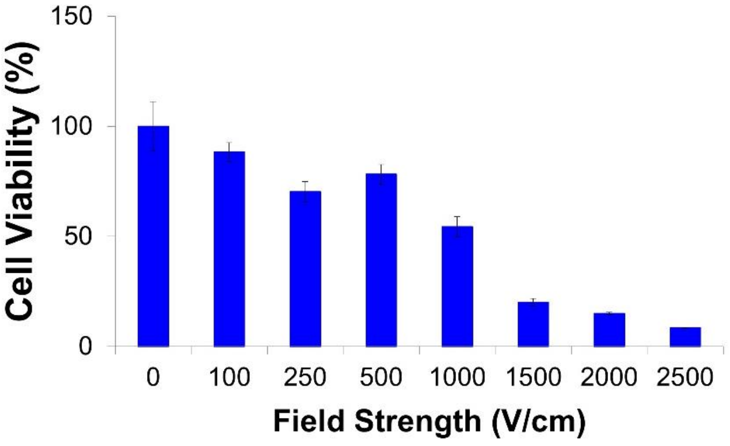

Figure S1. Cytotoxicity of electroporation at various field strengths in immortalized human vascular endothelial RF24 cells 72 hours after treatment. No increased cell viability was observed in the RE range.
